# Global research trends on gut microbiota and metabolic dysfunction-associated steatohepatitis: Insights from bibliometric and scientometric analysis

**DOI:** 10.3389/fphar.2024.1390483

**Published:** 2024-07-11

**Authors:** Naqash Alam, Linying Jia, Ao Cheng, Honghao Ren, Yu Fu, Xinhua Ding, Ihtisham Ul Haq, Enqi Liu

**Affiliations:** ^1^ Laboratory Animal Center, School of Basic Medical Sciences, Health Science Center, Xi’an Jiaotong University, Xi’an, China; ^2^ Department of Neurobiology, School of Basic Medical Sciences, Health Science Center, Xi’an Jiaotong University, Xi’an, China

**Keywords:** NASH, gut microbiota, Citespace, VOSviewer, bibliometrix, visualization

## Abstract

**Background:**

Metabolic dysfunction-associated steatohepatitis (MASH) is an inflammatory subtype of metabolic dysfunction-associated steatotic liver disease (MASLD) has recently been proposed as a replacement term for NAFLD, a common, multifactorial and poorly understood liver disease whose incidence is increasing worldwide. In recent years, there has been increasing scientific interest in exploring the relationship between gut microbiota and MASH. To learn more about the gut microbiota in MASH, this study aims to provide a comprehensive analysis of the knowledge structure and research hotspots from a bibliometric perspective.

**Methods:**

We searched the Web of Science Core Collection for articles and reviews that covered the connections between gut microbiota and MASH over the last decade. The Online Analysis Platforms, VOSviewer, CiteSpace, the R tool “bibliometrix” were used to analyzed existing publications trends and hotspots.

**Results:**

A total of 4,069 documents related to the interaction between gut microbiota and MASH were retrieved from 2014 to 2023. The number of annual publications increased significantly over the last decade, particularly in the United States and China. The University of California-San Diego was the most productive institution, while researcher Rohit Loomba published the most papers in the field. Younossi ZM was ranked as the first co-cited author and largest contributor of highly cited articles in the field. Gastroenterology and hepatology were the most common specialty category. The most cited journal in the last decade was Hepatology. The Keyword Bursts analysis highlighted the importance of studying the association between gut microbiota and MASH, as well as related factors such as metabolic syndrome, insulin resistance, endotoxemia and overgrowth of gut bacteria. Keyword clusters with co-citation were used to illustrate important topics including intestinal permeability, insulin sensitivity and liver immunology. The most common keywords include insulin resistance, obesity, dysbiosis, inflammation and oxidative stress, which are current hotspots.

**Conclusion:**

Our analysis highlights key aspects of this field and emphasizes multiorgan crosstalk in MASLD/MASH pathogenesis. In particular, the central role of the gut-liver axis and the significant influence of gut microbiota dysbiosis on disease progression are highlighted. Furthermore, our results highlight the transformative potential of microbiota-specific therapies and cover the way for innovative healthcare and pharmaceutical strategies.

## Introduction

Nonalcoholic steatohepatitis (NASH) is an inflammatory form of nonalcoholic fatty liver disease (NAFLD) characterized by the presence of >5% hepatic steatosis in the absence of significant alcohol consumption or other underlying causes (Chalasani et al., 2018). NASH is characterized by hepatic steatosis associated with hepatocyte inflammation and injury (ballooning) (Chalasani et al., 2018), and possibly leading to progressive fibrosis. The degree of liver fibrosis is an important predictor of hepatic morbidity and mortality (Younossi et al., 2011; Ekstedt et al., 2015; Dulai et al., 2017; Vilar-Gomez et al., 2018). While both NAFLD and NASH can trigger progressive liver fibrosis, NASH has a higher propensity for this progression (Wong et al., 2010; Younossi et al., 2016). It is estimated that 25% of people worldwide have NAFLD and 2%–6% have NASH (Younossi et al., 2016). The prevalence of NAFLD in the United States estimated to be 37%; approximately 8% of NAFLD patients have advanced liver fibrosis (Ciardullo and Perseghin, 2021). NASH is becoming more common, but more alarming is the disproportionate increase in individuals with severe fibrosis, hepatocellular cancer and hepatic decompensation expected in model studies (Estes et al., 2018). NASH is associated with a poor prognosis because patients are frequently asymptomatic or have nonspecific symptoms (Sheka et al., 2020). According to the 2018 American Association for the Study of Liver Diseases (AASLD) guidelines, NAFLD can be diagnosed non-invasively by radiographic assessment; However, liver biopsy is currently required to differentiate NASH from NAFLD (Chalasani et al., 2018). An assessment method for the histological features of NAFLD (the NAFLD activity score (NAS)) was developed to track changes during therapy studies, since the diagnosis of NASH is determined by the presence and pattern of certain histological abnormalities in liver biopsy (Hashimoto et al., 2013). In general, NAS of ≥5 correlates strongly with NASH diagnosis, while most clinical trials have an inclusion criterion of NAS ≥4. There are currently no approved therapies to treat NASH (Chalasani et al., 2018). Unsurprisingly, NASH is now one of the most common reasons for liver transplantation around the world (Holmer et al., 2018; Calzadilla-Bertot et al., 2019; Parrish et al., 2019; Shingina et al., 2019; Younossi, 2019). Therefore, a new nomenclature was introduced. Patients with steatosis or steatohepatitis and the presence of at least one cardiometabolic risk factor are now diagnosed as metabolic dysfunction associated steatotic liver disease (MASLD) or steatohepatitis (metabolic dysfunction associated steatohepatitis (MASH)) (Rinella et al., 2023). Over 95% of patients with NAFLD have MASLD, while the small group of non-MASLD NAFLD patients requires further study to understand the underlying causes. The new nomenclature offers a solution for the co-existence of metabolic risk factors with other causes of liver disease, most notably the co-existence with excessive alcohol consumption (metabolic and alcohol-related liver disease (MetALD)) (Zeng et al., 2024).

The human microbiota is estimated to comprise of nearly 10^13 to 10^14 microbial cells, which corresponds to a ratio of approximately 1:1 to human cells (Sender et al., 2016). These numbers are derived primarily from the total number of bacterial cells in the colon, which harbors the densest microbial population, estimated at 3.8 × 10^13 bacteria (Sender et al., 2016). These gut microbiotas thrive symbiotically in the digestive tract and interact with the host through metabolites, microbiota-associated molecular patterns (MAMPs), membrane vesicles and other mechanisms (Albillos et al., 2020; Agirman and Hsiao, 2021). Due to the far-reaching effects of microbial communities on the gut and distal organs, microbiome research has expanded to nearly all systems of the human body in recent decades (Foster, 2022). A growing body of research shows that the gut microbiota controls the gut microenvironment to modulate immune system responses, essential for maintaining physiological homeostasis. However, dysbiosis refers to changes in the structure or diversity of the gut microbiota caused by genetic or environmental variables such as dietary traits or drugs like antibiotics or nonsteroidal anti-inflammatory drugs (Noureddin and Sanyal, 2018; Parthasarathy et al., 2020). In addition, the gut microbiota is involved in balancing pro-inflammatory and anti-inflammatory signals, contributing to inflammation and progression of MASH (Kolodziejczyk et al., 2019).

Over the past decade, clinical research found that the primary manifestation of these changes is a decrease in bacterial diversity in the gut microbial flora of MASLD patients (Wang et al., 2021). At the same time, the gut vascular barrier (GVB) is damaged as a result of the high fat diet (HFD), which also leads to microbial group disorders in MASLD mice. This damage promotes the infiltration of pathogen-associated molecular patterns (PAMPs), which exacerbates inflammatory responses (Mouries et al., 2019). The major bile acid receptor in the liver and small intestine is the farnesoid X receptor (FXR). According to studies, inhibition of FXR signaling in the gut could reduce production of liver ceramide and fatty acids, which could reduce liver lipid accumulation and improve HFD-induced MASLD (Cai et al., 2022). In addition, many studies suggest that the gut microbiota and its metabolites play important role in the development and progression of MASLD, which are important targets of MASLD treatment (Safari and Gérard, 2019a). Recent work provided clear evidence that dysbiosis promotes MASLD through various mechanisms including dysfunction of bile acid metabolism, reduction of short-chain fatty acids (SCFA), inhibition of fasting-induced adipocyte factor (FIAF) production, increase in intestinal permeability, changes of intestinal motility (Leung et al., 2016). Recently, bibliometric analysis and data visualization have received significant attention in the biomedical field data and the growing number of freely available bibliometric tools (Chen, 2006; van Eck and Waltman, 2010). This study is based on the visual analysis using CiteSpace and VOSviewer software to clarify the research situation and trend on MASH and gut microbiota in the last decade, thus providing new ideas and broad perspective and valuable information for ongoing studies to other researchers.

## Materials and methods

### Data source search strategies

**FIGURE 1:**
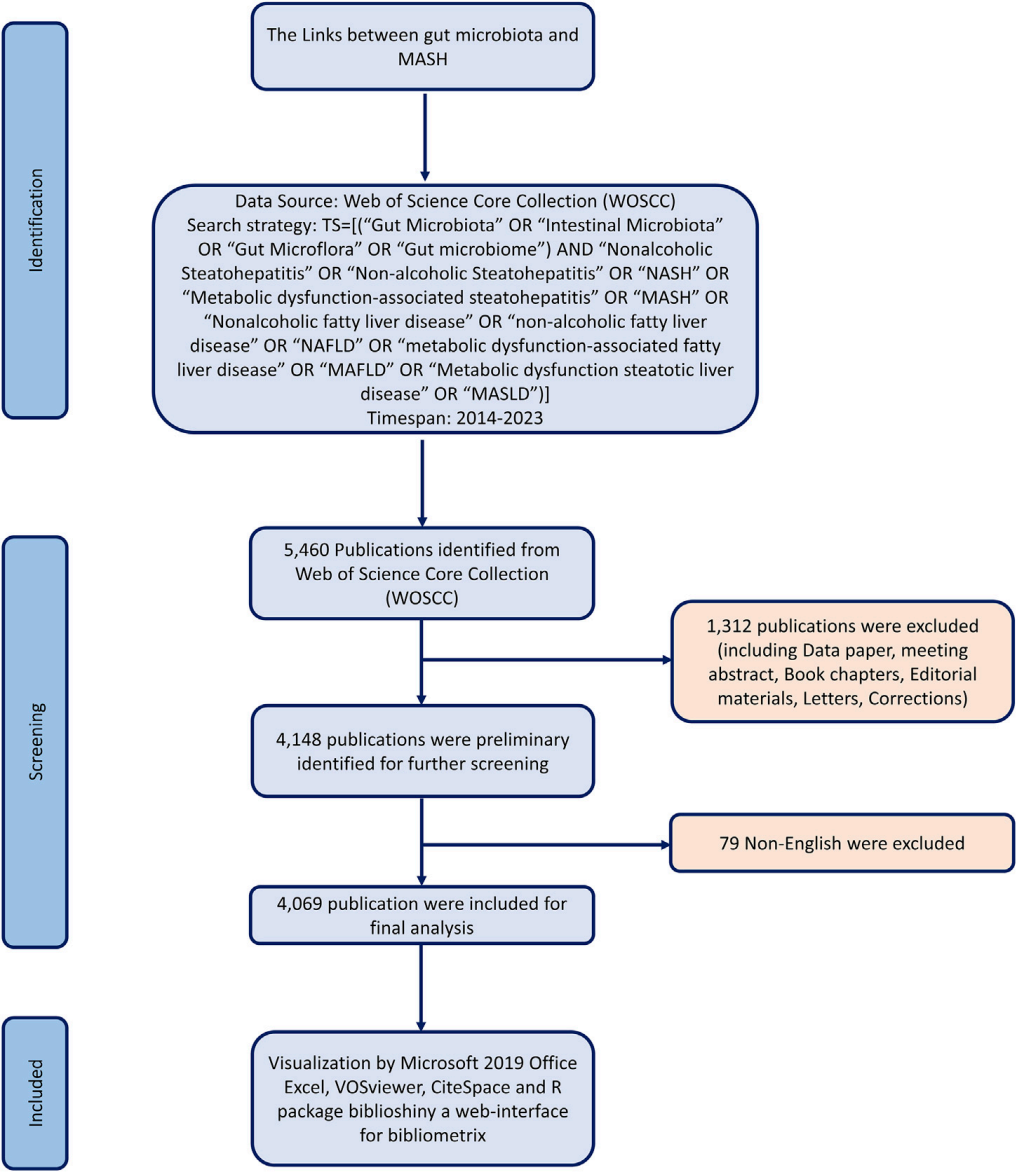
Flowchart of the search and selection process, including the criteria for inclusion and exclusion of publications.

Literature was extracted from the Web of Science Core Collection database and downloaded on 30 December 2023. To avoid biases from database updates, we completed all data extraction and data downloads on the same day. All potentially relevant publications were collected by the following search strategy: TS = [(“Gut Microbiota” OR “Intestinal Microbiota” OR “Gut Microflora” OR “Gut microbiome”) AND “Nonalcoholic Steatohepatitis” OR “Non-alcoholic Steatohepatitis” OR “NASH” OR “Metabolic dysfunction-associated steatohepatitis” OR “MASH” OR “Nonalcoholic fatty liver disease” OR “non-alcoholic fatty liver disease” OR “NAFLD” OR “metabolic dysfunction-associated fatty liver disease” OR “MAFLD” OR “Metabolic dysfunction steatotic liver disease” OR “MASLD”)] AND Language = English and Document Type = Article and Review Article. The raw data was then downloaded from WoSCC as text files containing full records. After conducting the primary data search, two researchers reviewed all documents individually to ensure their relevance to the topic of this study. Ultimately, 4,069 documents were analyzed in our study. Figure 1 illustrates the detailed screening process.

### Data extraction and collection

All 4,069 retrieved documents were downloaded with “full record and cited references” and exported in plain text or tab-delimited format for bibliometric tool analysis. Subsequently, statistical analysis was carried out with Microsoft Excel 2019 on bibliometric key figures such as the annual number of publications and citations, countries/regions, institutions, authors, journals, keywords and research fields. Journal Impact Factor (JIF) and subject category quartile rankings were taken from the 2022 Journal Citation Report (JCR, http://clarivate.com/products/web-of-science). JCR divides all journals within the same discipline into four categories based on the value of JIF, with the top 25% belonging to Q1, the top 25%–50% to Q2 and so on. Other bibliometric information obtained from “citation report” function of WoSCC included the sum of time cited and average number of citations.

### Bibliometric analysis

We converted all WoSCC data that met the criteria into TXT format and imported into Online Analysis Platform of Literature Metrology (https://bibliometric.com/app), R-package Bibliometrix, CiteSpace V5.8 R3 (Drexel University, Philadelphia, PA, United States) and VOSviewer 1.6.15 (Leiden University, Leiden, Netherlands) for further analysis to describe all literature features on the relationship between gut microbiota and MASH (Chen, 2006; van Eck and Waltman, 2010). Figure 1 showed a flowchart illustrating the search and selection process, including inclusion and exclusion criteria of publications. The Bibliometrix R package was used to generate word cloud of the top 100 keywords. In addition, cooperation between countries/regions and between institutions was analyzed using the VOSviewer software. CiteSpace, the most popular and widely used bibliometric visualization tool (Chen, 2006), was used to generate various figures to help understand the current status of the links between gut microbiota and MASH research and to generate potential hotspots in this field, such as co-citation analysis, citation burst, clustered networks of co-cited references and keywords with the strongest citation bursts.

## Results

### Quantity and trends analysis of published papers

**FIGURE 2:**
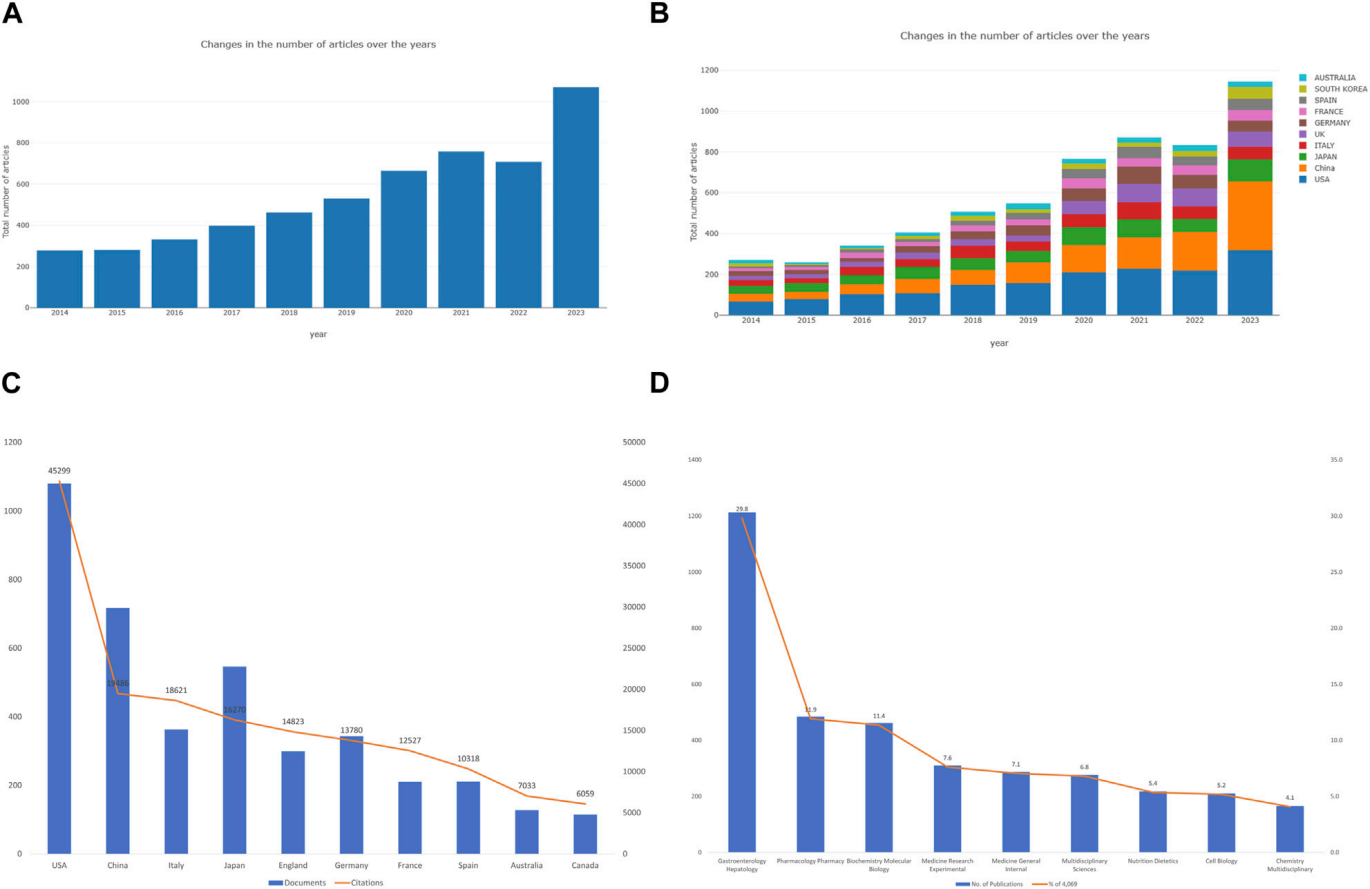
Analysis of publications on the associations between gut microbiota and MASH from 2014 to 2023. **(A)** Annual research publications and growth patterns on gut microbiota and MASH, data exported from WoSCC. **(B)** Annual research publications and growth trends on gut microbiota and MASH, data exported from an online literature metrology analysis platform. **(C)** Top 10 countries by total number of citations. **(D)** Top 10 research areas by number of publications.

A total of 4,069 publications between 2014 and 2023 met the criteria for inclusion in the Web of Science core collection database. We excluded 1,391 publications (non-English publications, meeting abstracts, data papers, proceeding papers, book chapters). As shown in Figure 2A, studies of the relationships between gut microbiota and MASH were categorized into two time periods. The early-stage publication trend (2013-2019) maintained a fluctuating growth, whereas the number of publications (2020-2023) grew at nearly triple the rate of the former, notably in the last 3 years, indicating that the intricate linkage is largely established came into the gut microbiota and MASH gained worldwide attention. In terms of publication types, there were a total of 3,042 research articles and 1,072 review articles. It appears that gut microbiota and MASH is a popular area of research that attracted a lot of attention from academics year over year. The Online Analysis Platform of Bibliometrics (http://biblimetric.com/) was used to quantify the number of publications from different countries and regions. This analysis aimed to identify the leading contributors to research on the connections between gut microbiota and MASH over the past decade. The bar chart in Figure 2B showed the number of publications from the top ten countries over a 10-year period. Surprisingly, the United States dominated all other countries in the field of gut microbiota and MASH, while China emerged as the leading country since 2016 and its publication production maintained an increasing growth trend.

### Analysis of country/region and academic institution distribution

**FIGURE 3:**
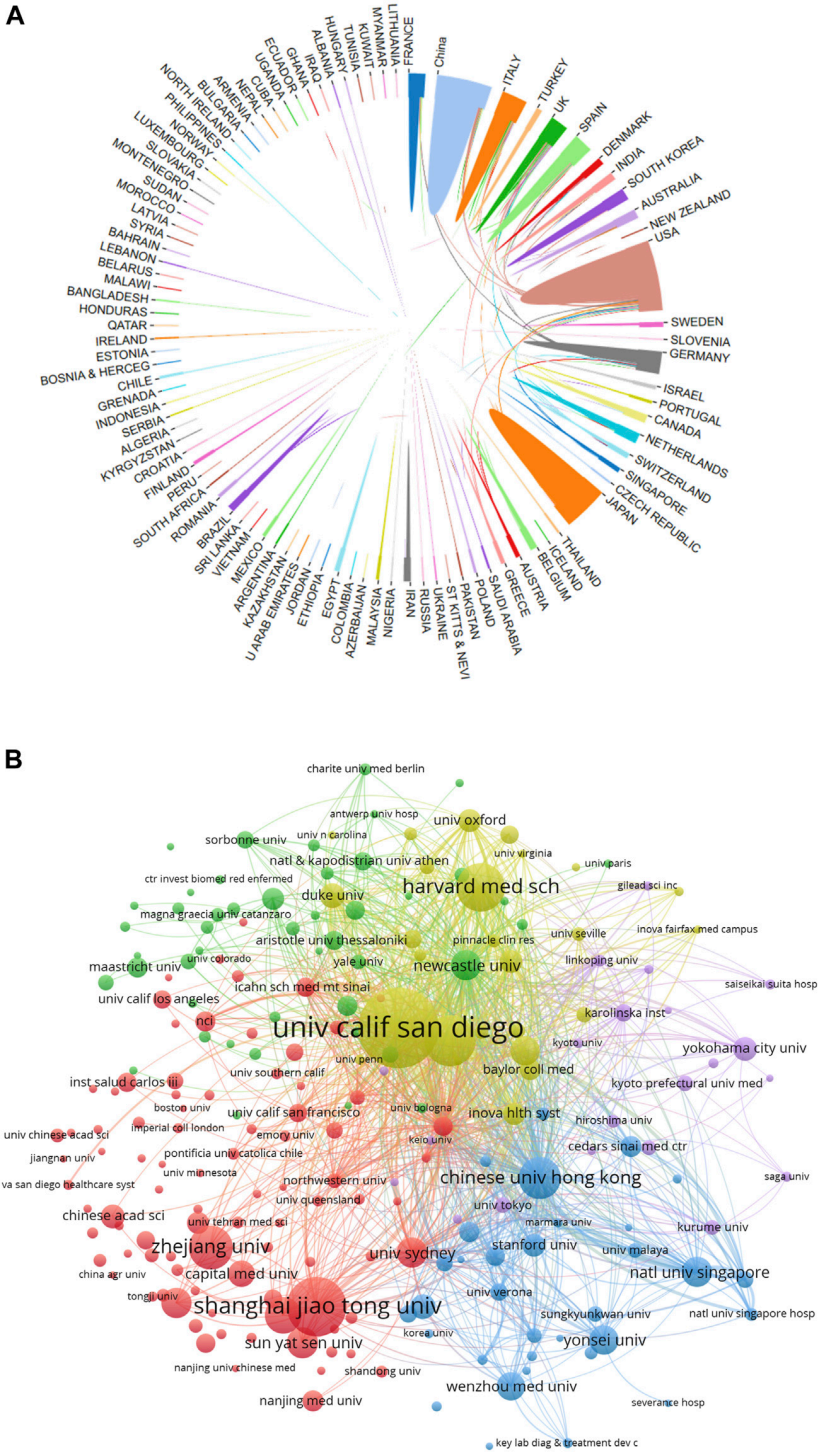
**(A)** Collaborative relationships 97 countries/regions studying gut microbiota and MASH from 2014 to 2023, analyzed using an online literature metrology platform. **(B)** VOSviewer network map of the top 100 most productive institutions in gut microbiota and MASH research. Cluster size reflects the number of publications, while line thickness indicates the degree of inter-institutional collaboration.

In total, 4,069 documents were published by 97 countries and regions between 2014 and 2023. We analyzed the collaborative relations between these countries using the bibliometrics online analysis platform. Figure 3A showed the scientific collaboration network between countries and regions researching the connection between gut microbiota and MASH. Each circle represented a country/region, and the connections demonstrated the strength of international collaboration. The results showed that the United States (1,079/26.51%) had the highest number of publication followed by China (716/17.59%) and Japan (545/13.39%) were the most frequently involved in international cooperation. In terms of total citations, the United States were cited 44,966 times, followed by China (19,153) and Italy (18,288) Figure 2C. Table 1 showed the top ten countries in terms of average number of citations. The top three countries with the highest average number of citations per article were France (average of 59.65 citations per article), Australia (52.76) and Italy (50.52), each article from these countries were cited more than 50 times on average. The average number of citations per article in each country was calculated by dividing the total number of citations by the total number of publications for that country (total citations of each country/total publications of each country). In addition, all of these literature were assigned to different research areas based on WoS subject categories. Figure 2D showed the top ten research areas, sorted by number of publications. From an institutional perspective, 4,751 institutions focused on the research area of gut microbiota and MASH. Regarding the collaborative relationships between them, we listed the top 100 institutions (with >20 articles) in the institutional co-occurrence analysis Figure 3B. It was observed that institutions with the highest number of publications had very close collaborative relationships with each other. Among them, the University of California-San Diego published the most papers (262 publications) followed by Shanghai Jiao tong university (194), Virginia commonwealth university (167), Harvard Medical School (159), Zhejiang University (143) and Chinese university Hong Kong (138). The rankings based on citation parameters fluctuated to varying degrees compared with the output rankings.

**TABLE 1:** Top 10 countries ranked by the highest average number of citations per article from a dataset of 4,069 articles on gut microbiota and MASH research (based on average citation count).

Country	Documents	Citations	Avg. Number of citations	Total link strength
France	210	12,527	59.65	4,792
Australia	127	6,700	52.76	2,763
Italy	362	18,288	50.52	7,230
Canada	114	5,726	50.23	2,184
England	298	14,490	48.62	7,788
Spain	210	9,985	47.55	3,959
United States	1,079	44,966	41.67	15,270
Germany	342	13,447	39.32	6,050
Japan	545	15,937	29.24	5,257
China	716	19,153	26.75	8,329

### Analysis of authors and co-cited authors

**FIGURE 4:**
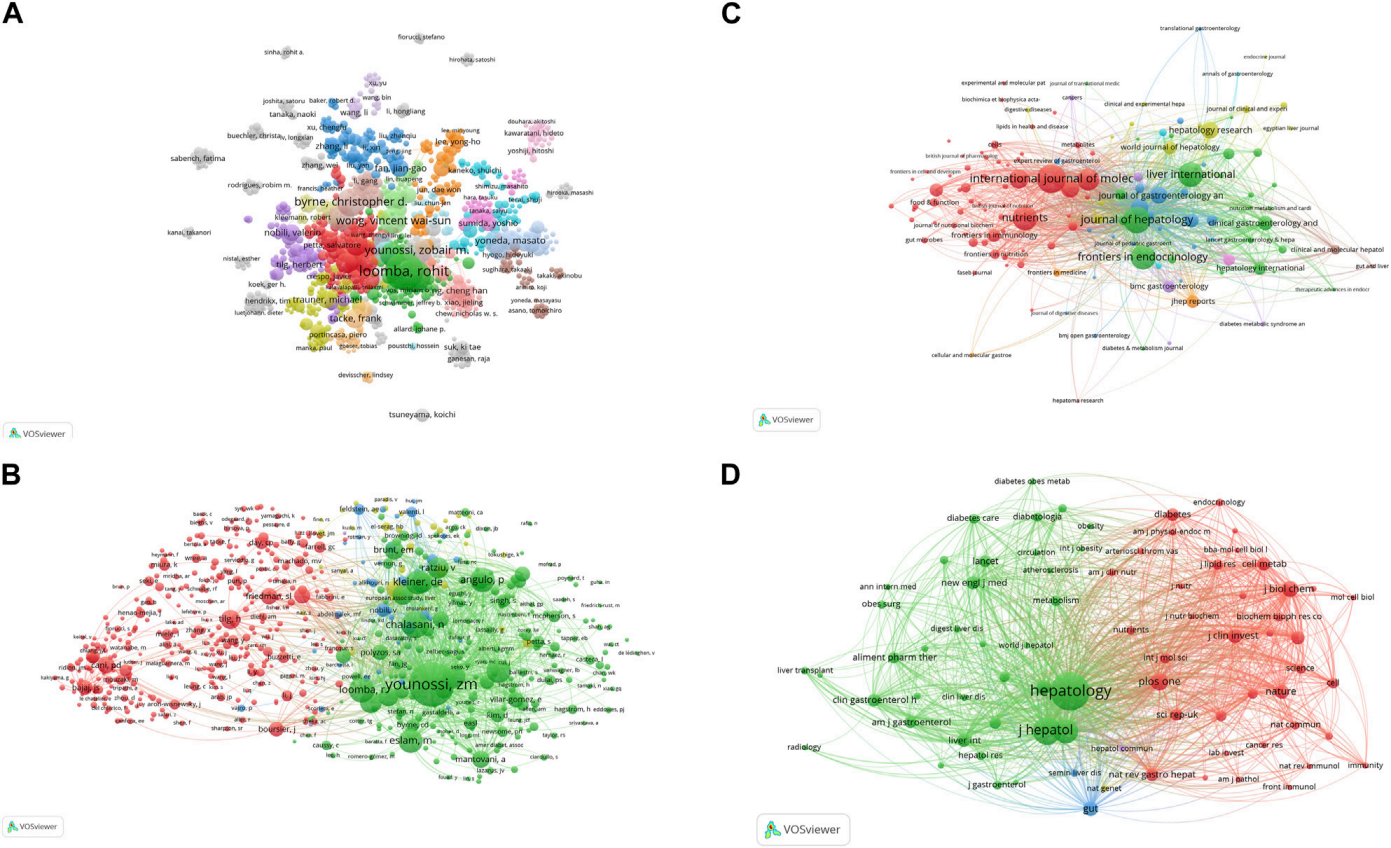
VOSviewer network analysis of authorship and co-citation in gut microbiota and MASH research. **(A)** Overlay visualization of authors. **(B)** Density visualization of co-cited authors. Each circle represents an author, with connections indicating collaboration. Font size correlates with the number of published articles. **(C)** Citation network and clusters of the top 100 most cited journals. **(D)** Network visualization of the most cited journals based on VOSviewer analysis.

A total of 23,350 authors participated in research into gut microbiota and MASH over the past decade. Among the top 10 authors, six authors had each published 30 or more papers (Supplementary Table S1). Visual mapping provided clear information about collaborative relationships, which helps in finding potential collaborators. The font size depended on the number of publications published by specific authors. When it came to the connection between the gut microbiota and MASH scientific community, these authors were the most prolific. We created a collaborative network of authors with at least five published publications Figure 4A. Loomba Rohit., Sanyal Arun J., Byrne Christopher D., Younossi Zobair M., Wong Vincent Wai-Sun., Targher Giovanni., Harrison Stephen A., Nakajima Atsushi., Anstee Quentin M., and George Jacob had the biggest nodes because they published the most relevant publications. In contrast to the largely independent networks in institutions, it was remarkable that the majority of the authors, especially the productive ones, chose to establish stable cooperation networks. Among the 23,350 co-cited authors, 10 authors were co-cited more than 500 times (Supplementary Table S2). The most frequently co-cited author was Younossi ZM (n = 5,505), followed by Chalasani N (n = 2,377), Loomba R (n = 2,129), Angulo P (n = 2012) and Kleiner DE (n = 1972). Authors with a minimum of 100 co-citations were filtered to map co-citation network graphs. Figure 4B showed the ongoing collaborative efforts of various co-cited authors, indicating a dynamic network of scholarly interactions and partnerships. In order to gain additional insights, the figure represented a complex network of scientific commitment and cooperation efforts by the cited authors.

### Analysis of journals and co-cited journals

Journals are a powerful factor in the presentation of scientific research results and the dissemination of knowledge. Understanding all relevant journals in their field is a challenge for many researchers, who often struggle to select the most suitable issue and target journals for their studies. Over the past decade, 911 scientific journals collectively published 4,069 documents. The VOSviewer software was used to analyze the citation network among these journals, with a threshold set to more than eight total publications to create visible maps for 100 journals Figure 4C. The top 10 most-cited journals related to the associations between gut microbiota and MASH were listed in Table 2. Hepatology had the highest number of citations (33,940) over the past 10 years, followed closely by Journal of Hepatology (33,325), Gastroenterology (19,092), Nature reviews gastroenterology and hepatology (15,900), World journal of gastroenterology (15,900), Gut (8,538), International Journal of molecular sciences (7,859), Nutrients (7,737), Clinical gastroenterology and hepatology (6,687), Liver International (6,171).The paper published in Gastroenterology received the highest average citations per article (329.1). In addition, half of these journals are from the United States.

**TABLE 2:** Leading scientific highly cited journals in gut microbiota and MASH research: A citation analysis.

Journals	Documents	Citations	Avg. Citations	Country	If	JIF quartile
Hepatology	136	33,940	249.5	United States	14.0	Q1
Journal of Hepatology	259	33,325	128.6	Netherland	25.7	Q1
Gastroenterology	58	19,092	329.1	United States	29.4	Q1
Nature Reviews Gastroenterology and Hepatology	55	15,900	289.0	United States	65.1	Q1
World Journal of Gastroenterology	191	9,272	48.5	United States	4.3	Q2
Gut	58	8,538	147.2	England	24.4	Q1
International Journal of Molecular Sciences	282	7,859	27.8	Switzerland	5.6	Q1
Nutrients	223	7,737	34.6	Switzerland	5.9	Q1
Clinical Gastroenterology and Hepatology	110	6,687	60.7	United States	12.6	Q1
Liver International	230	6,171	26.8	Denmark	6.7	Q1

By summarizing key information and mapping the co-citation networks of influential journals, researchers can pinpoint the core journals in gut microbiota and MASH research. This method allows researchers to select the optimal journals for manuscript submissions. Most relevant studies were published in Q1 or Q2 journals among the top ten most productive journals. As shown in Table 3, among the top 10 co-cited journals, five journals were cited more than 4,000 times, Hepatology (co-citation = 48,612) was the most cited journal, followed by Journal of Hepatology (co-citation = 28,845), Gastroenterology (co-citation = 21,508), Plos One (co-citation = 11,658), Gut (co-citation = 9,837) (co-citation = 4,101). In addition, Gastroenterology had the highest impact factor (IF = 29.4), followed by Journal of Hepatology (IF = 25.7). Journals with a minimum co-citation of 500 were filtered to map the co-citation network. As shown in Figure 4D, Hepatology showed positive co-citation relationships with Journal of Hepatology. Research related to the connections between gut microbiota and MASH published in these journals had great potential for citation and attention. Therefore, scholars may have considered these priority journals in the past, and the scholarly outcomes of these journals also deserved special attention for providing the latest advances in this field.

**TABLE 3:** Leading scientific co-cited journals in gut microbiota and MASH research: A citation analysis.

Journals	Citations	Total link strength	Country	If	JIF quartile
Hepatology	48,612	4,084,008	United States	14.0	Q1
Journal of hepatology	28,845	2,649,560	Netherland	25.7	Q1
Gastroenterology	21,508	2,036,556	United States	29.4	Q1
Plos one	11,658	1,167,113	United States	3.7	Q2
Gut	9,837	1,003,880	England	24.4	Q1
Nature	9,080	998,839	England	64.8	Q1
World journal of Gastroenterology	8,215	754,754	United States	4.3	Q2
Journal of Biological Chemistry	7,818	877,781	United States	4.8	Q2
Clinical Gastroenterology and Hepatology	7,815	692,957	United States	12.6	Q1
Nature Reviews Gastroenterology&Hepatology	7,457	668,045	United States	65.1	Q1

**FIGURE 5:**
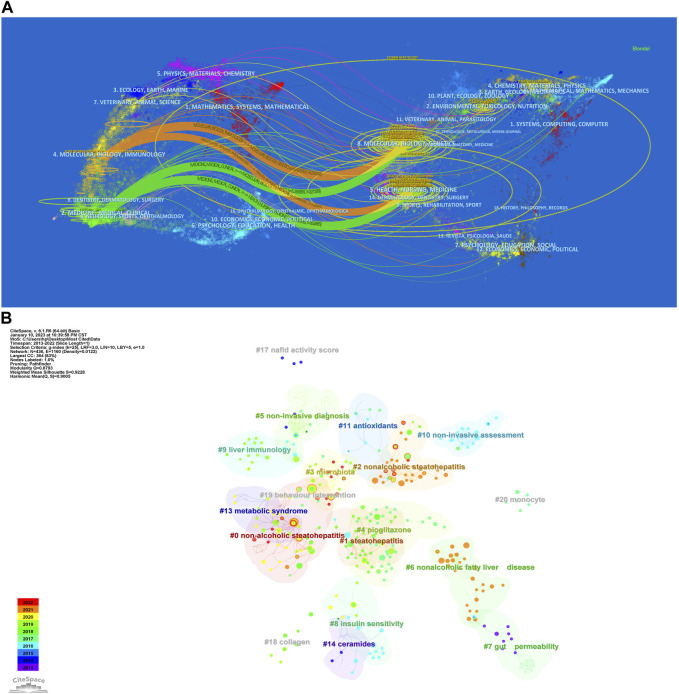
**(A)** Dual-map overlay of journals related to gut microbiota and MASH generated by CiteSpace. **(B)** CiteSpace-generated clustered networks showing co-citation relationships between the investigated references and the 4,069 citing articles.

The dual-map of CiteSpace illustrated the development of research across multiple areas. As depicted in Figure 5A, it showed citing articles on the left, cited articles on the right, and the citation relationships were indicated by the colored curved path in the middle. The four orange or green citation paths indicated that research in molecular biology, genetics and health, nursing and medical journals was frequently cited by molecular biology/immunology and medical/medical/clinical journals. At the same time, disciplines such as Veterinary Medicine/Animal Sciences/Natural Sciences, Ecology/Earth/Marine Sciences, Physics/Materials/Chemistry, Environmental Sciences/Toxicology/Nutrition, and Psychology/Educational/Social Sciences, displayed in the margins of the overlay plots were also involved in the research of gut microbiota and MASH, which demonstrated to some extent that researchers conducted multidisciplinary and collaborative studies in this area.

### Analysis of number of citations

The number of citations is a major indicator of the impact of an article in a research field. The number of citations of these 4,069 documents were counted and ranked and the top 10 are listed in Table 4. Henao-Mejia et al. from Yale University published the most cited article in Nature (Henao-Mejia et al., 2012), which was cited 1,696 times. This study highlighted the critical role of the gut microbiota in the pathogenesis of systemic autoinflammation and metabolic diseases by showing how the NLRP6 and NLRP3 inflammasomes negatively control the development of MASH and metabolic syndrome by altering them. The second and third articles were published in the Lancet and Nature with 1,374 and 1,273 citations, respectively (Yoshimoto et al., 2013; Neuschwander-Tetri et al., 2015). Both confirmed that gut bacteria play a role in the development of insulin resistance and MASH. The article in 10th place was published in Nature Reviews Gastroenterology and Hepatology in 2020 with 260 citations. This study provided a broad overview of microbiome signatures for human MASLD and examined issues related to distinguishing these signatures from underlying metabolic disorders (Aron-Wisnewsky et al., 2020).

**TABLE 4:** The top 10 most cited articles from a dataset of 4,069 retrieved articles on gut microbiota and MASH research (sorted by citation frequency).

Rank	Title	First author	Journal	Year	Cited frequency	DOI
1	Inflammasome-mediated dysbiosis regulates progression of NAFLD and obesity	Henao-Mejia, J	Nature	2012	1,696	10.1038/nature10809
2	Farnesoid X nuclear receptor ligand obeticholic acid for non-cirrhotic, non-alcoholic steatohepatitis (FLINT): a multicentre, randomised, placebo-controlled trial	Neuschwander-Tetri, BA	Lancet	2015	1,384	10.1016/S0140-6,736(14)61,933-4
3	Obesity-induced gut microbial metabolite promotes liver cancer through senescence secretome	Yoshimoto, S	Nature	2013	1,273	10.1038/nature12347
4	Metabolic profiling reveals a contribution of gut microbiota to fatty liver phenotype in insulin-resistant mice	Dumas, ME	PNAS	2006	881	10.1073/pnas.0601056103
5	Probiotics and antibodies to TNF inhibit inflammatory activity and improve nonalcoholic fatty liver disease	Li, ZP	Hepatology	2003	755	10.1053/jhep. 2003.50048
6	The Severity of Nonalcoholic Fatty Liver Disease Is Associated with Gut Dysbiosis and Shift in the Metabolic Function of the Gut Microbiota	Boursier, J	Hepatology	2016	681	10.1002/hep.28356
7	Obeticholic acid for the treatment of non-alcoholic steatohepatitis: interim analysis from a multicentre, randomised, placebo-controlled phase 3 trial	Younossi, ZM	Lancet	2019	497	10.1016/S0140-6,736(19)33,041-7
8	Intestinal Microbiota in Patients with Nonalcoholic Fatty Liver Disease	Mouzaki, M	Hepatology	2013	466	10.1002/hep.26319
9	Gut Microbiota Profiling of Pediatric Nonalcoholic Fatty Liver Disease and Obese Patients Unveiled by an Integrated Meta-Omics-Based Approach	Del Chierico, F	Hepatology	2017	357	10.1002/hep.28572
10	Gut microbiota and human NAFLD: disentangling microbial signatures from metabolic disorders	Aron Wisnewsky, J	Nature Reviews Gastroenterology and Hepatology	2020	260	10.1038/s41575-020-0269-9

### Analysis of document co-citation and clustered network

**FIGURE 6:**
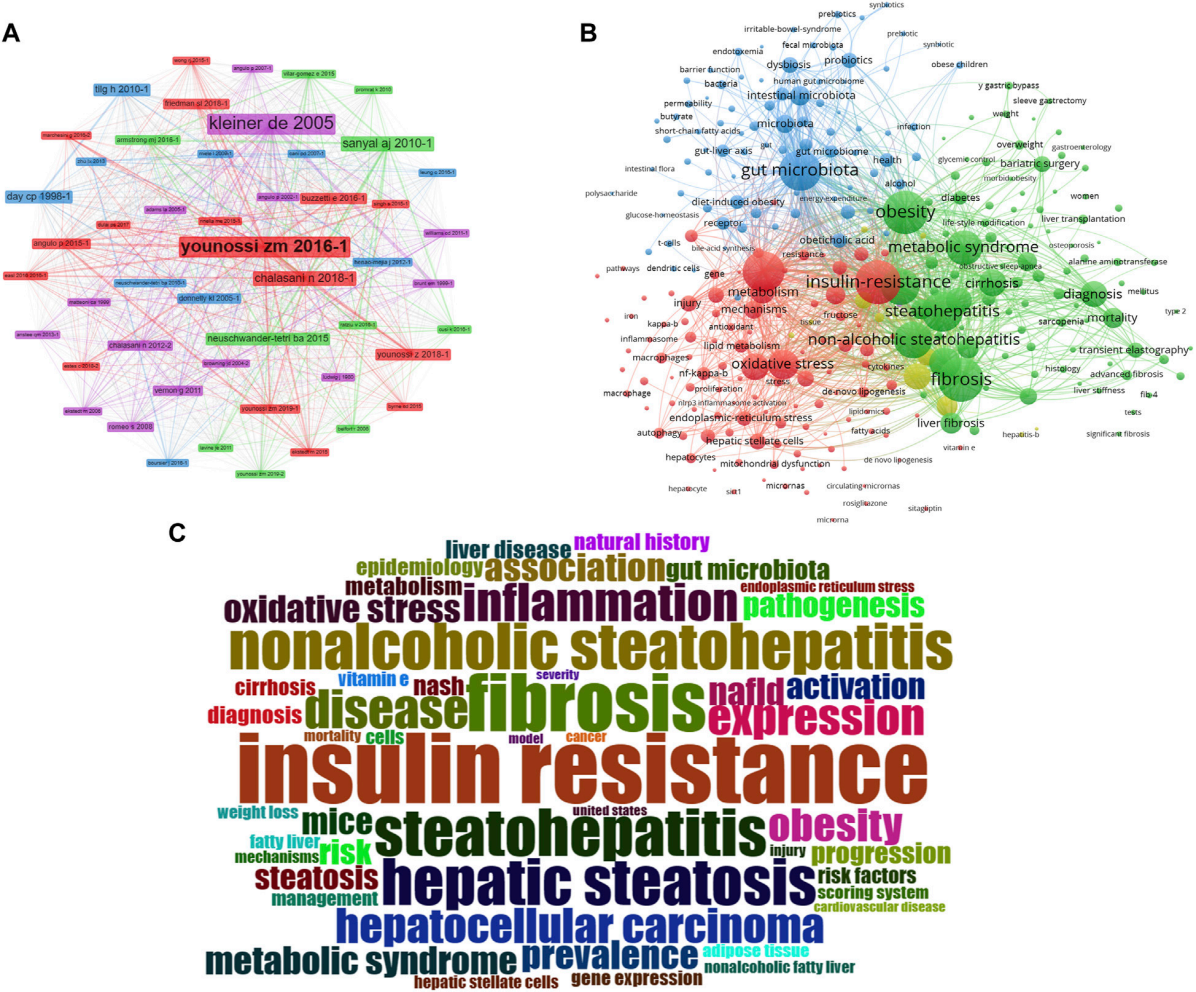
**(A)** Co-citation map of 119,803 references connections in gut microbiota and MASH research, with the filter option showing only the largest connected component. **(B)** Clustering analysis of high-frequency keywords. **(C)** Word cloud illustrates the top 100 most frequently used terms in studies exploring the relationship between gut microbiota and MASH. (Panel A and C generated using R package Bibloshiny, while Panel B was created using VOSviewer).

In the 4,069 publications retrieved, 119,803 references were cited. References that are frequently cited in conjunction with other articles are considered fundamental to a particular area of ​​research. By analyzing co-citations of cited references, researchers discovered the background and knowledge base of gut microbiota and MASH research. Figure 6A depicted a map of co-citation references on the links between gut microbiota and MASH. Each node represented a reference, with links between nodes indicating that these publications were cited as references in the same article among the 4,069 articles retrieved. The size of the node was related to citation frequency, and the line thickness indicated the relationship with co-cited papers. Furthermore, the nodes that appeared redder on the color bars indicated that these works had been frequently cited in recent years, reflecting their importance in the field. The top ten references of 4,069 publications sorted by citation frequency, were listed in Table 5.

**TABLE 5:** The top 10 references with the highest co-citation frequencies among 4,069 retrieved articles on gut microbiota and MASH research.

Rank	Title	First author	Journal	Year	Citations	DOI
1	Design and validation of a histological scoring system for nonalcoholic fatty liver disease	Kleiner DE	Hepatology	2005	1,061	10.1002/HEP.20701
2	Global epidemiology of nonalcoholic fatty liver disease-Meta-analytic assessment of prevalence, incidence, and outcomes	Younossi ZM	Hepatology	2016	645	10.1002/HEP.28431
3	The diagnosis and management of nonalcoholic fatty liver disease: Practice guidance from the American Association for the Study of Liver Diseases	Chalasani N	Hepatology	2018	428	10.1002/HEP.29367
4	Pioglitazone, vitamin E, or placebo for nonalcoholic steatohepatitis	Sanyal AJ	NEJM	2010	357	10.1056/NEJMOA0907929
5	Steatohepatitis: a tale of two “hits"	Day CP	Gastroenterology	1998	351	10.1016/S0016-5,085(98)70,599-2
6	The diagnosis and management of non-alcoholic fatty liver disease: practice Guideline by the American Association for the Study of Liver Diseases, American College of Gastroenterology, and the American Gastroenterological Association	Chalasani N	Hepatology	2012	326	10.1002/HEP.25762
7	Evolution of inflammation in nonalcoholic fatty liver disease: The multiple parallel hits hypothesis†	Tilg H	Hepatology	2010	326	10.1002/HEP.24001
8	Global burden of NAFLD and NASH: trends, predictions, risk factors and prevention	Younossi ZM	Nature Reviews Gastroenterology and Hepatology	2018	323	10.1038/NRGASTRO. 2017.109
9	Liver Fibrosis, but No Other Histologic Features, Is Associated with Long-term Outcomes of Patients with Nonalcoholic Fatty Liver Disease	Angulo P	Gastroenterology	2015	303	10.1053/J.GASTRO. 2015.04.043
10	Systematic review: the epidemiology and natural history of non-alcoholic fatty liver disease and non-alcoholic steatohepatitis in adults	Vernon G	Alimentary Pharmacology and Therapeutics	2011	297	10.1111/J.1365-2036.2011.04724.X

We applied CiteSpace in order to further analyze the co-cited references, with the following parameters set: time slicing (2014-2023), years per slice (1), node type (cited reference), selection criteria (k = 25), and no pruning. Clusters were generated using keywords retrieved from the references in Figure 5B. The value of the cluster numbers showed the intensity of the focus on the cluster topic within the discipline. The lower the cluster score, the greater the attention. According to the results, the cluster structure was significant and highly reliable, with an overall modularity Q of 0.8793 and an average silhouette of each cluster greater than 0.9. Clusters #0-3, #6-9, #13 and #18 examined the triggering factors for the connections between gut microbiota and MASH. Clusters #5 and #10 explored the diagnostic and quantitative issues of gut microbiota and MASH. Cluster #4, #11 and #14 explored the mechanisms and treatment strategies of the gut microbiota and MASH.

**FIGURE 7:**
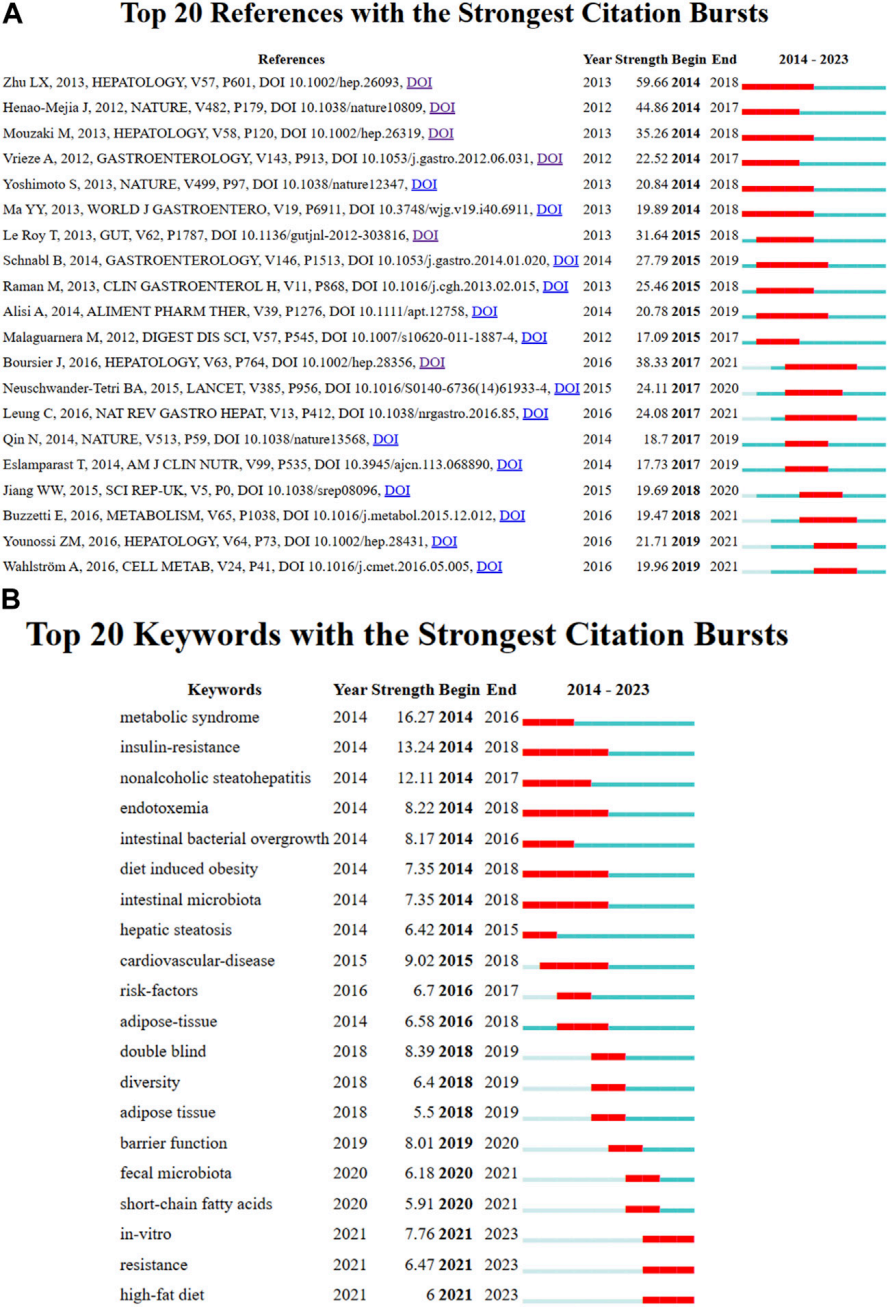
**(A)** Analysis of the reference with the highest burst strength among 4,069 citing articles on gut microbiota and MASH research published from 2014 to 2023. References marked in red denote a significant increase in usage frequency during this period, while blue indicates a comparatively lower frequency. **(B)** Keywords with the highest burst strength. Keywords highlighted in red denote a notable increase in usage, while blue denotes a less prominent period of usage. (The figure created using CiteSpace).

Burst detection of cited references represented a shift in research focus in a subject. In CiteSpace we set the parameters minimum duration = 2, g = 1 and screened 20 references with the strongest citation bursts, indicating their importance for gut microbiota and MASH- related areas Figure 7A. The blue line represented the period from 2014 to 2023, while the red line represented the duration of the burst references. Among these references, the strongest citation burst starting with the article published by Zhu LX et al. (2013) observed heightened alcohol-producing bacteria in MASH microbiomes and elevated blood ethanol levels, indicating a potential role in MASH pathogenesis through alcohol metabolism-induced oxidative stress. The distinctive gut microbiome composition in MASH emerges as a potential intervention target or diagnostic marker for the disease. Henao-Mejia J et al. (2012) demonstrated in various mouse models that changes in gut microbiota configuration due to inflammasome deficiency correlate with increased liver steatosis and inflammation. This process involves the influx of TLR4 and TLR9 agonists into the portal circulation, amplifying hepatic tumor necrosis factor (TNF)-α expression and driving MASH progression. Mouzaki M et al. (2013) identified a diet- and BMI-independent inverse association between the presence of MASH and the percentage of Bacteroidetes in stool, suggesting an integral role of intestinal microbiota in MASH development. Boursier J et al. (2016) established a link between MASLD severity and gut dysbiosis, with *Bacteroides* associated with MASH and Ruminococcus with significant fibrosis. Gut microbiota analysis enhances classical predictors, unveiling potential targets for pre−/probiotic therapies. Le Roy T et al. (2013) demonstrated that variations in microbiota composition determine the response to a high-fat diet (HFD) in mice, emphasizing the crucial role of gut microbiota in MASLD development, irrespective of obesity.

### Keyword analysis and burst detection

The keyword co-occurrence network facilitates the identification of research hotspots and trends within a field. In this study, we used VOSviewer software to perform keyword analysis, the minimum number of occurrences of a keyword was set as 5, and a total of 665 keywords were extracted Figure 6B. Further cluster analysis of the keywords revealed three clusters, indicating three research directions and study topics. Cluster 1 (red) was the most components including insulin resistance, oxidative stress, metabolisms, endoplasmic reticulum, autophagy, macrophage, *de novo* lipogenesis, etc. Followed by cluster 2 (green) including Obesity, metabolic syndrome, fibrosis, cirrhosis, diabetes, bariatric surgery, y gastric bypass, sleeve gastrectomy, life style modification, liver transplant, etc. Cluster 3 (blue) mainly including gut microbiota, dysbiosis, gut-liver axis, probiotics, prebiotics, symbiotic, endotoxemia, barrier function, permeability, butyrate, SCFA, diet-induce-obesity, polysaccharides, etc. Cluster one and two primarily represented the pathophysiology of the gut microbiota in MASH and several therapeutic targets under development. Clusters three terms primarily indicated diseases associated with the gut microbiota in MASH and the link between gut microorganisms and metabolic diseases.

Keyword burst detection was another method that helped to catch research hotspots quickly. We also used CiteSpace to visualize the keywords co-occurrence network on gut microbiota and MASH from 2014 to 2023, focusing on the top 20 with the most keyword bursts Figure 7B. According to our results, several keywords have emerged as critical research interests in this research field. Notably, the keyword burst of metabolic syndrome, which began in 2014 and lasted for 3 years with a strength of 16.27, was found to be a precursor to MASH. This is a significant finding as metabolic syndrome is a risk factor for MASH and its early detection and management can prevent the progression of the disease. Additionally, the second strongest keyword burst of 13.24 was observed for insulin resistance, which occurred between 2014 and 2018 and was associated with the concept of MASH. This highlights the importance of exploring the relationship between these two conditions to better understand their interaction. Furthermore, endotoxemia was found to be a critical keyword burst with a strength of 8.22 between 2014 and 2018. Endotoxemia, the presence of endotoxins in the blood, is involved in MASH pathogenesis. Elevated endotoxin levels in the blood are due to both intestinal and hepatic factors. Scientific research demonstrated a link between endotoxemia and intestinal dysbiosis, indicating an imbalance in the composition of the intestinal microbiota.

Intestinal bacterial overgrowth, with a keyword burst strength of 8.17 between 2014 and 2016, underscored significant alterations in the intestinal microbiota. These microbiota changes were linked to hepatic fat accumulation, emphasizing the crucial role of intestinal microbiota in the pathogenesis of liver diseases. Moreover, evidence from transient elastography and liver biopsy studies reveals a strong association between intestinal bacterial overgrowth and the increased prevalence of MASH, highlighting the pivotal role of intestinal microbiota in liver disease progression. Finally, barrier function emerged as another important area of research interest with a strength of 8.1 between 2019 and 2020. Disturbances in intestinal barrier function are associated with the occurrence and progression of liver diseases, including MASH. The main mechanisms by which the intestinal barrier influences MASH development include changes in the epithelial layer and reduced integrity of the intracellular junction. The top 100 keywords related to the connections between gut microbiota and MASH studies are shown in a word cloud in Figure 6C. Frequency and font size are positively related.

### Three-field plot and evolution themes in gut microbiota and MASH research

**FIGURE 8:**
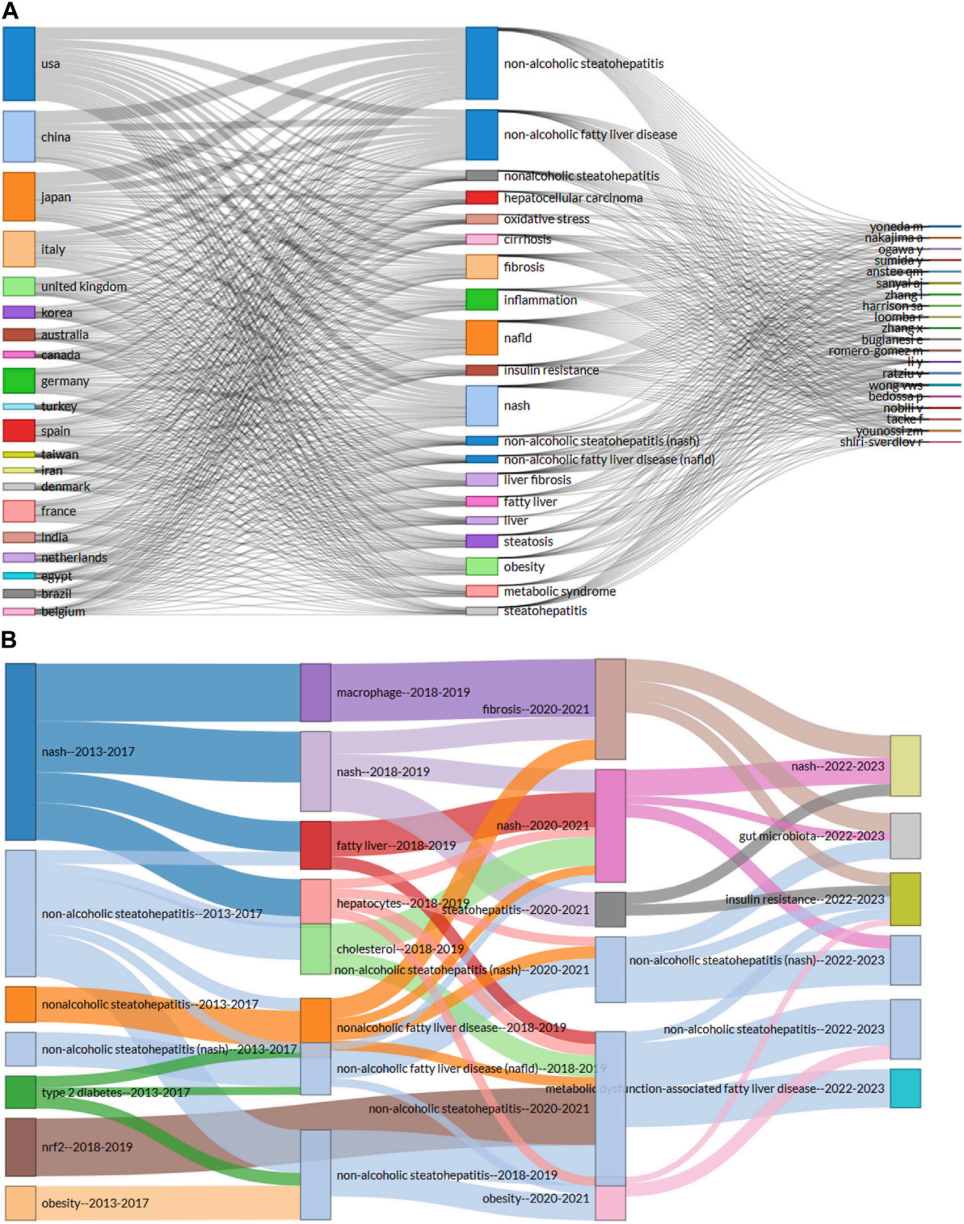
**(A)** A three-field plot (Sankey diagram) illustrates prolific authors, their countries and areas of interest represented by keywords in the field of gut microbiota and MASH research. **(B)** Thematic evolution across three phases of gut microbiota and MASH research, depicted by lines connecting nodes that reflect the evolving focus of the research topic. Line width indicates the number of common keywords, with thicker lines indicating greater thematic importance. (Figure generated using R package Bibloshiny).

Three-field plot provided a clear visualized and concise representation of prolific authors, their countries and their areas of interest presented with keywords related to gut microbiota and MASH as shown in Figure 8A. The left column represented the countries, the middle column represented the most frequently used keywords by these authors and the right column showed the names of the researchers who contributed to this area. The frequency of occurrences of the keyword forms was referred to as the theme. The height of the boxes along with the thickness of the connecting lines enhanced the interaction and connectedness between countries. The United States had the highest author affiliations, followed by China. In the same order Japan had the next highest number of authors followed by Italy and the United Kingdom. It was observed that the thickness of the line leading from countries to authors indicated the greatest contributions made to the area of ​​ gut microbiota and MASH.

A Sankey diagram (also known as a Sankey energy diffluence diagram) was created Figure 8B. The Sankey diagram was used in this article to visualize the thematic evolution of gut microbiota and MASH research over time. It showed the flow of multiple topics in gut microbiota and MASH research and provided quantitative information on thematic flows, orientations, and relationships. In Figure 8B, subjects were represented as rectangles. The size of a rectangle depended on the number of keywords within the theme. The lines connecting the rectangles showed the evolutionary flow of the research theme. The thematic temporal continuity between adjacent time zones was represented by the connections between rectangles. The thickness of a line indicated how many words were repeated and reflected the close relationship between topics. Each line color was used to distinguish between research themes in Figure 8B. The entire research process in gut microbiota and MASH was analyzed using the thematic evolution map to track the progression and extinction of themes, highlighting evolutionary divergence from evolutionary trends. The evolutionary path map and state of each time period showed the ongoing development of gut microbiota and MASH research over different time periods. The analysis revealed a clear evolution of research themes over time and highlighted the dynamic interplay and divergence between thematic relationships. Furthermore, the combination, transmission and renewal of themes highlighted the complex and evolving nature of gut microbiota and MASH research.

## Discussion

### General information

In this bibliometric study, we identified 4,069 articles related to the relationship between gut microbiota and MASH research published in WoSCC from 2014 to 2023. The total number of publications showed a stable growth trend, reflecting a significant increase in research output on various topics within the field. Using an online bibliometric analysis, we systematically reviewed the publication trends in gut microbiota and MASH research from different perspectives over the last decade. Researchers interested in gut microbiota and MASH can efficiently obtain a comprehensive overview and identify current research hot spots and emerging trends in the field. The results of the annual publications in this area showed a continuous and steady upward trend over the past decade. Figure 2B indicated that the United States and China were the two leading countries that contributed significantly to gut microbiota and MASH research. On the one hand, the United States consistently contributed a significant proportion of publications over the last decade. On the other hand, although Chinese researchers entered the field later than other nations, they quickly established themselves as prominent contributors to gut microbiota and MASH research. In addition, increasing international collaboration has become an irreversible trend, and this type of collaboration is more conducive to the production of high-quality research results. In terms of cooperation between countries/regions as shown in Figure 3A, the United States remained at the top, followed by China in terms of cooperation relationships with other countries/regions. Although there were cooperative links in some countries, the breadth and depth of collaboration between institutions was not ideal.

The top 10 institutions were universities, suggesting that universities were the most common research groups. Three of the top 10 institutions were from the United States (University California San Diego, Virginia Commonwealth University, and Harvard Medical School); Therefore, the United States is the main domain in this field. Three of the top 10 institutions were from China (Shanghai Jiao Tong University, Zhejiang University and Chinese University Hong Kong) and two institutions were from the United Kingdom (Newcastle University, University of Oxford). According to the results of this analysis, the three countries have numerous large research groups in this field. Additionally, among the top ten institutes by publication count, the University of California-San Diego ranked first and its contributions to this area have been focused in recent years Figure 3B. As a result, these outcomes indicated extensive research and significant scientific potential in the field of gut microbiota and MASH.

This study identified notable research contributions from distinguished authors in the field. From the perspective of the top ten authors who have contributed significantly to the study of gut microbiota and MASH over the past decade, such as Loomba Rohit, Sanyal Arun J, Byrne Christopher D, Younossi Zobair M, and Wong Vincent Wai-Sun published the most publications in this area. In the study on non-alcoholic fatty liver disease, Sanyal Arun J et al. Integrating problems with chronic liver disease, steatosis, metabolic syndrome and insulin resistance. Steatohepatitis research involves subjects such as nonalcoholic hepatitis and liver biopsy. Hepatocellular carcinoma, clinical trials, hepatology and liver transplantation are all components of cirrhosis research (Sanyal et al., 2010). Loomba Rohit et al. study designs that complement human intervention studies with mechanistic work in mice that have been humanized in multiple respects, including genetic, immunological and microbiome, are making rapid progress towards clinical applications. This is especially true for study designs that focus on the relationship between the microbiome and liver disease (Tripathi et al., 2018). The main interest of Ratziu Vlad et al. was the gut microbiome, metagenome associated with liver disease, the role of microbial products and metabolites in liver disease. Targeting the gut microbiota can be a preventive or therapeutic approach to treating MASLD, as the gut bacteria play a role in the development of obesity and steatosis (Burz et al., 2021).

In the top 10 most active journals that published documents on the links between gut microbiota and MASH research, the Hepatology journal (IF = 14.0) was cited most frequently (33,940). The Journal of Hepatology (IF = 25.7) was the top journal in the field of the liver containing all aspects of liver structure, function and disease. Furthermore, Gastroenterology papers (IF = 29.4) received the highest average citation (289.0). Of the top 10 journals four were from the United States, two from the United Kingdom, and the remaining four from the Netherlands, Switzerland, Denmark, and Australia, reflecting the fact that the United States primarily provided a communication platform for research into the relationship between gut microbiota and MASH. Regarding the co-cited journals, we found that most of them were high impact Q1 journals. Noticeably, these journals represented high-quality international platforms that supported research on gut microbiota and MASH. In addition, current research on gut microbiota and MASH was mostly published in Hepatology, Journal of Hepatology, Gastroenterology, Plos One, World Journal Gastroenterology, Journal of Biological Chemistry, Gut and Nature-related journals. Very few studies were published in clinically related journals, suggesting that most of the current research is still at the basic research stage.

Based on the total number of citations by each country, the United States and China were undoubtedly the two most influential countries on gut microbiota and MASH over this decade. The main reason was that the United States and China ranked first and second in the world in terms of the number of articles published. However, the situation changed when considering at the average number of citations per article. France, Australia and Italy recorded the highest average number of citations per article, indicating the high average quality of articles from these three countries. Among the top 10 publications with the most citations, half were clinical research and the other half were basic research. One of the most cited publications addressed basic research on the effect of microbiota on insulin resistance and metabolic syndrome in the development of MASLD. This highlighted that the interaction between microbiota and systemic metabolism is a focus of research in the field of gut microbiota and MASH (Li et al., 2003; Dumas et al., 2006; Henao-Mejia et al., 2012; Mouzaki et al., 2013; Yoshimoto et al., 2013; Neuschwander-Tetri et al., 2015; Boursier et al., 2016; Del Chierico et al., 2017; Younossi et al., 2019; Aron-Wisnewsky et al., 2020). A co-cited reference is a source cited by numerous other publications and considered a cornerstone of research in a field. In this bibliometric analysis, we identified the 10 co-cited references with the highest number of co-citations to evaluate the scientific basis of gut microbiota and MASH. Kleiner et al. published the most frequently co-cited study in 2005, this study aimed to develop and validate a trait-based semi-quantitative scoring system to be used in clinical and natural history studies of MASLD (Kleiner et al., 2005). In addition, recent study collectively expands our understanding of liver diseases and possible therapeutic strategies. Dynamic contrast-enhanced photoacoustic imaging (PAI) is emerging as a promising tool for assessing liver fibrosis progression in mice and correlates well with histological assessments (Lv et al., 2021). The top 10 co-cited references discussed the following topics: a systematic review and meta-analytic technique, the global incidence, prevalence, disease progression, and burden of MASLD and MASH, inflammation, risk factors and prevention, which constitute the foundation for this research (Sanyal et al., 2010; Vernon et al., 2011; Angulo et al., 2015; Younossi et al., 2016; Chalasani et al., 2018). Moreover a meta-analysis on genetic studies showed that TM6SF2 rs58542926 is associated with a lower risk of MASLD, while MBOAT7 rs641738 shows no significant association (Xia et al., 2019). Furthermore, molecular studies demonstrated downregulation of miR-199 in hepatocellular carcinoma (HCC), where it directly suppresses XBP1, providing new insights into HCC pathogenesis (Lou et al., 2018). Therapeutically, TO901317 showed promise in HCC treatment by modulating LXRα and metabolic pathways, while studies highlighted the protective role of reduced glutathione against alcohol-induced liver injury. Overall, these results highlighted the diverse approaches to understanding and potentially treating liver disease (Xiong et al., 2019).

### Research hotspots and trend

The most valuable information that bibliometric analysis can provide is the knowledge base and research frontier in a particular field, which can be reflected by literature co-citation, co-occurrence analysis and burst detection respectively. The bibliometric analysis of keywords in this study mainly focused on three primary research areas: mechanisms, influencing factors, and diagnostic approaches (Figure 6B). This analysis revealed the striking features and research topics in this area. The progression of MASLD to MASH is a complicated multi-factorial process the exact mechanism is obscure. The “two-hit hypothesis” is currently widely accepted as a plausible explanation by the general public (Day and James, 1998). The core premise is that hepatic steatosis and insulin resistance constitute the “first hit,” which causes triglycerides to build up in liver cells (Sumida et al., 2013). The “second hit” occurs when liver dysfunction, including hepatocyte inflammation, liver fibrosis and cirrhosis develops as a result of the combined action of inflammatory factors, oxidative stress and endoplasmic reticulum stress (Kawano and Cohen, 2013). The “two-hit hypothesis” has been replaced in more recent years by the “multiple parallel hit hypothesis”. Consistent with the multiple parallel hit hypothesis, MASH is caused by a combination of genetic variation, insulin resistance, aberrant lipid metabolism, endoplasmic reticulum stress, mitochondrial dysfunction and gut microbiota (Takaki et al., 2013; Boursier and Diehl, 2016). Taken together, these mechanisms describe the key pathological processes essential to the progression of MASH. Notably, past research figures prominently in the scientific investigation landscape as evidenced by the prevalence of commonly occurring keywords that predominantly focus on the risk factor. At the same time, factors predisposing to MASH, such as obesity and metabolic syndrome, as well as factors associated with hepatocellular carcinoma (HCC) particularly cirrhosis were consistently identified as frequently co-occurring keywords.

A major significant contributor to the onset of MASLD is systemic insulin resistance (Parthasarathy et al., 2020). Insulin resistance triggers the activation of lipolysis in adipose tissue and leads to the release of free fatty acids (FFAs) into the bloodstream. The FFAs are absorbed by the liver and undergo *de novo* lipogenesis, leading to the accumulation of triglycerides (TGs) in hepatocytes, a characteristic feature of MASLD (Ferro et al., 2020; Ng et al., 2022). The abnormal accumulation of TGs in hepatocytes, termed hepatic steatosis, marks the initial phase of MASLD and represents a crucial point in the disease progression (Ferro et al., 2020). Steatohepatitis categorized by hepatocyte ballooning, inflammation and liver fibrosis represents a complex progression from simple steatosis to steatohepatitis. This transition involves complicated mechanisms such as oxidative stress, mitochondrial dysfunction and impaired lipid metabolism (Flessa et al., 2022).

Oxidative stress plays a central role in MASLD pathogenesis (Ferro et al., 2020). The excessive accumulation of FFAs in hepatocytes triggers the production of reactive oxygen species, leading to oxidative damage and inflammation, thus perpetuating disease progression (Parthasarathy et al., 2020). Mitochondrial dysfunction contributes significantly to the pathophysiology of fatty liver and adds a critical layer to the complex landscape of metabolic dysregulation. Impaired mitochondrial function and increased mitochondrial fission are notable features observed in MASLD, leading to increased oxidative stress and hepatocyte damage (Zhou et al., 2018). Endoplasmic reticulum (ER) stress represents another central mechanism involved in the pathogenesis of MASLD (Song and Malhi, 2019; Flessa et al., 2022). ER stress occurs when the ER exceeds its protein folding capacity, leading to activation of the unfolded protein response. Prolonged ER stress is associated with hepatocyte damage, inflammation and fibrosis in the context of MASLD (Song and Malhi, 2019; Flessa et al., 2022).

Inflammation plays a critical role in the progression of MASLD from steatosis to steatohepatitis (Parthasarathy et al., 2020). Macrophages play a crucial role in liver inflammation, as resident liver macrophages are activated in obesity conditions and modulate inflammatory pathways (Lefere and Tacke, 2019). In addition, there is a significant increase in the infiltration of macrophages and other immune cells in the liver. This leads to the production of inflammatory cytokines that induce insulin resistance in hepatocytes and contribute to the development of type 2 diabetes-related diseases, including MASLD and MASH (Obstfeld et al., 2010). Activation of pattern recognition receptors (PRRs) by pathogen- and danger-associated molecular patterns (PAMPs and DAMPs) triggers immune cell activation and inflammatory changes in MASLD (Parthasarathy et al., 2020; De Muynck et al., 2021). Inflammatory cytokines such as interleukin-6 (IL-6) and tumor necrosis factor-alpha (TNF-α) are increased in MASLD and play a role in hepatocyte damage and fibrosis (Bruneau et al., 2021; De Muynck et al., 2021). Dysregulation of lipid metabolism represents another key feature of MASLD. Aberrations in lipid uptake, synthesis, oxidation and export contribute to lipid accumulation in hepatocytes, thereby driving the progression of hepatic steatosis (Perla et al., 2017). Dysregulation of lipid metabolism can also lead to the formation of toxic lipid metabolites such as diacylglycerols and ceramides, which can worsen inflammation and cause hepatocyte damage (Perla et al., 2017).

The interaction between the gut and liver plays a crucial role in the development and progression of MASLD, with dysbiosis contributing to insulin resistance, inflammation and oxidative stress. Dysbiosis of the intestinal microbiota changes in intestinal barrier function and metabolic endotoxemia are key factors that significantly contribute to the development of MASLD (Ni et al., 2020). Gut dysbiosis marked by an imbalance in the composition of the gut microbiota has been implicated in the progression of obesity and MASLD (Ferro et al., 2020; Bruneau et al., 2021). Numerous studies have investigated the molecular mechanisms underlying gut dysbiosis in individuals with metabolic disorders. In obesity, gut dysbiosis is associated with alterations in the diversity and composition of the gut microbiota. Dysbiosis can lead to increased dietary energy production, metabolic disorders and inflammation associated with obesity (Boursier et al., 2016). In obesity, dysbiosis is characterized by a reduction in beneficial bacteria and an elevation in detrimental bacteria (Jiang et al., 2015). Similar to individuals with obesity, gut dysbiosis has been linked to the pathogenesis of MASLD.

Nevertheless, several bacterial species were linked with MASLD in individuals, with the abundance of species such as Proteobacteria, Enterobacteria, *Escherichia* (Fukui, 2019), or *Bacteroides* being higher in MASH patients than in corresponding healthy individuals (Boursier et al., 2016). Patients with MASLD had a lower abundance of Bacteroidetes, Ruminococcaceae and Faecalibacterium prausnitzii compared to healthy individuals, while concurrently showing increased prevalence of Prevotella, Porphyromas, *Lactobacillus*, *Escherichia* and *Streptococcus* species (Adolph et al., 2018; Wang et al., 2019). MASH patients had decreased fecal Bacteroidetes levels and increased *Clostridium* coccoides levels (Mouzaki et al., 2013). However, patients with liver cirrhosis had elevated levels of Veillonella, Megasphaera, Dialister, Atopobium and Prevotella (Yu et al., 2016).

Another line of evidence supporting the pathogenesis of MASLD could be the strong correlation observed between gut microbiota, type 2 diabetes mellitus (T2DM) and insulin resistance. T2DM is associated with excessive production of pro-inflammatory cytokines, including interleukin (IL)-1α, IL-6, IL-10 and IL-2 (Randeria et al., 2019). The gut microbiota plays a role in modulating the inflammatory response through the secretion of anti-inflammatory cytokines. For example, Roseburia intestinalis promotes the production of IL-22 an anti-inflammatory cytokine, while attenuating insulin resistance and the development of diabetes (Wang et al., 2014). The gut microbiota is intricately linked to T2DM as it has the capacity to regulate insulin clearance (Cunningham et al., 2021). Furthermore, insulin resistance is associated with increased intestinal permeability in the presence of dysbiosis, regardless of whether it is related to obesity (Scheithauer et al., 2020). Overall, gut microbiota dysbiosis contributing to the development of T2DM and insulin resistance may position MASLD as a hepatic manifestation of systemic insulin resistance (Sakurai et al., 2021).

Bariatric surgery is considered the most effective treatment modality for achieving long-term weight loss and resolving obesity-related comorbidities, including T2DM, MASLD, cardiovascular disease and reduced mortality rates (Courcoulas et al., 2020). Bariatric surgery contributes significantly to improving biochemical and histological parameters in individuals with MASLD (Baldwin et al., 2019). Several studies suggest that Roux-en-Y gastric bypass surgery (RYGB) alters not only the composition of the microbiota but also its microbial functions. Consequently, increased protein degradation, increased functional annotations and improved fatty acid utilization are often observed following RYGB surgery (Ilhan et al., 2017). This led to the hypothesis that there is a reduction in energy yield from food after gastric bypass surgery.

The gut-liver axis describes the interaction between the liver, gut and gut microbiota (Albillos et al., 2020). It plays a crucial role in the development and progression of MASLD (Safari and Gérard, 2019b). The importance of a disrupted gut-liver axis in the development of liver disease has only recently come to light through improved understanding of the gut microbiome, gut barrier function and the role of bile in gut-liver communication (Albillos et al., 2020). Increasing evidence suggests that crosstalk between the gut microbiome, its metabolites, the immune system and the liver plays a crucial role in the development of ALD and MASLD. In both diseases, intestinal barrier dysfunction characterized by increased intestinal permeability, allows the portal influx of pathogen-associated molecular patterns (PAMPs) such as lipopolysaccharide (LPS or endotoxin) and microbiome-derived metabolites into the liver. This event triggers a proinflammatory cascade that exacerbates liver inflammation (Tilg et al., 2016). In the context of intestinal barrier dysfunction, the abundance of bacteria in the gut influences the level of PAMPs that translocate to the liver and bloodstream, ultimately affecting the severity of liver inflammation. The transition from compensated to decompensated chronic liver disease leads to damage to various levels of intestinal defense, leading to further functional worsening of the intestinal barrier (Albillos et al., 2020). Dysregulation of intestinal permeability in these liver diseases is associated with changes in the composition of the intestinal microbiota and disruption of tight junctions in the intestinal epithelium. These tight junctions are composed of several integral membrane proteins including zonula occludens (ZO), occludin, junctional adhesion molecule-A (JAM-A), claudins and there is an increased prevalence of small intestinal bacterial overgrowth (SIBO) (Bajaj, 2019; Martín-Mateos and Albillos, 2021). Disruption of the intestinal barrier followed by endotoxemia contributes to the progression and development of MASLD (Gudan et al., 2023).

**FIGURE 9:**
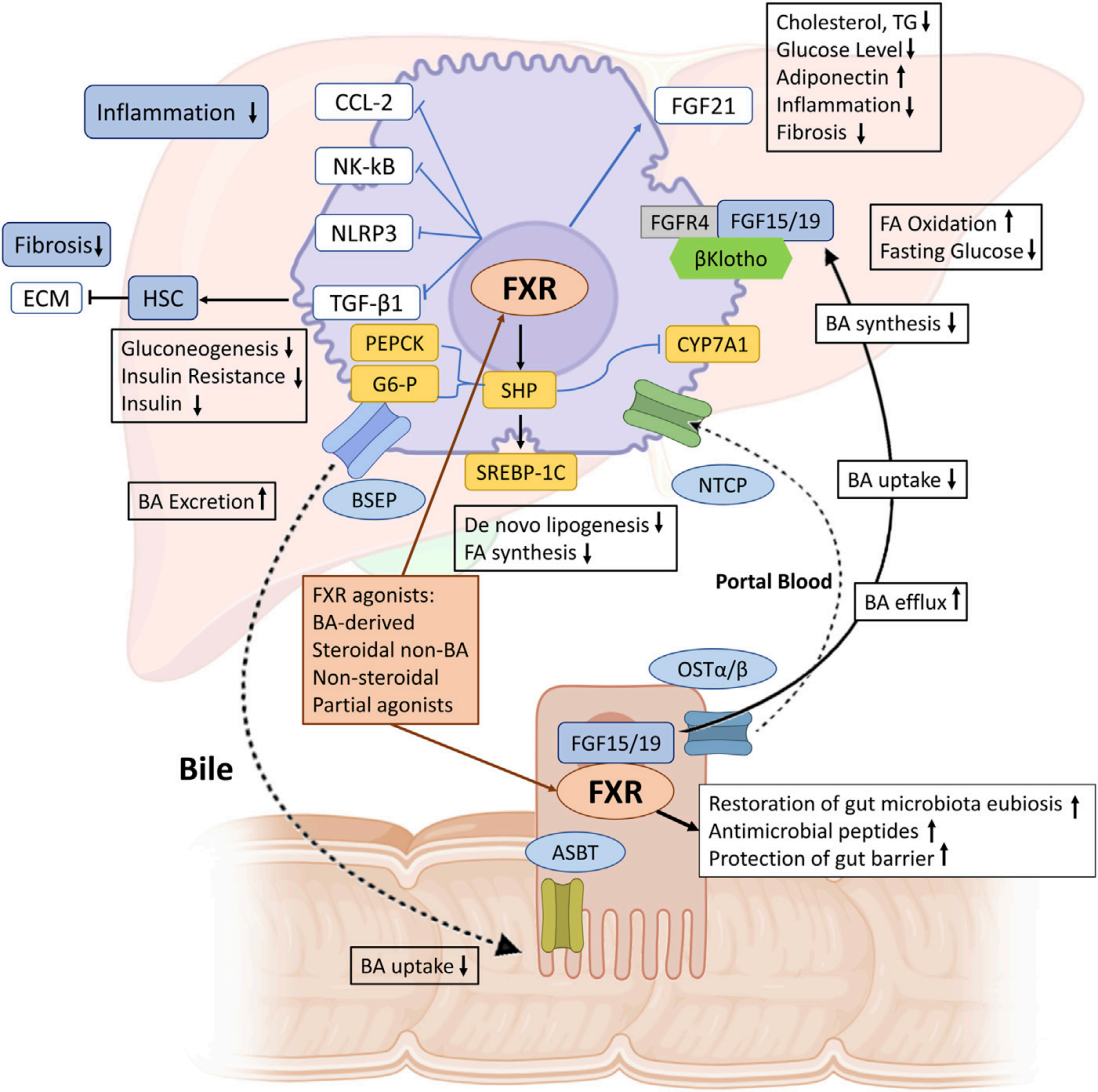
The role of FXR in maintaining BAs and metabolic homeostasis in MASH. BAs synthesized from cholesterol in the liver are excreted into bile via BSEP. Enterocytes mediate BA uptake and export into portal blood via ASBT and OSTα/β, respectively, with hepatocellular reuptake via NTCP completing the enterohepatic circulation. FXR activation increases bile secretion via BSEP and reduces BA synthesis and uptake via NTCP, thereby maintaining BA homeostasis. In MASH, hepatic FXR activation promotes FA oxidation, reduces *de novo* lipogenesis, inhibits inflammation (via NF-κB, NLRP3, and CCL-2), and reduces fibrosis (via TGF-β1 and ECM deposition by HSCs). FXR also upregulates FGF21, reduces cholesterol and triglyceride synthesis, lowers glucose levels, and increases adiponectin and adipocyte browning. FXR activation in the intestine increases FGF15/19, which binds to FGFR4/β-Klotho, increases fatty acid oxidation and glycogen synthesis, and inhibits CYP7A1 to reduce BA synthesis. Furthermore, FXR activation maintains the integrity of the intestinal barrier, prevents bacterial translocation, induces antibacterial peptides, and restores intestinal microbiota balance. FXR, farnesoid X receptor; BA, bile acid; BSEP, bile salt export pump; ASBT, apical sodium-dependent bile acid transporter; OSTα/β, organic solute transporter-α/β; NTCP, sodium taurocholate cotransporting polypeptide; NF-κB, nuclear factor j-light-chain enhancer of activated B cells; NLRP3, NOD-, LRR- and pyrin domain-containing protein 3; CCL-2, C–C motif chemokine ligand 2; TGF-β1, transforming growth factor-β1; ECM, extracellular matrix; HSC, hepatic stellate cell; FA, fatty acid; PEPCK, phosphoenolpyruvate carboxykinase; SREBP-1c, sterol regulatory element factor binding protein-1c; TG, triglyceride; FGF, fibroblast growth factor; SHP, small heterodimer partner. (Figure created with BioRender).

The metabolites produced by the gut microbiome are indispensable factors that can modulate the pathogenesis of MASLD and MASH. Most microbial metabolites arise primarily from the fermentation of carbohydrates and proteins. Short-chain fatty acids (SCFAs) represent one of the most common microbial metabolites derived from indigestible carbohydrates. SCFAs play a positive role in liver metabolism and are involved in the progression of MASLD. For example, a recent study identified a particular acetate derivative produced by a commensal microbe that can attenuate MASLD development by modulating FFAR2 signaling in the liver in high-fat-fed mice (Aoki et al., 2021). In addition, numerous studies have shown that another SCFA butyrate can alleviate MASLD by improving the gut microbiota, the tight junctions in the intestine, the expression of the glucagon-like peptide-1 (GLP-1) receptor in the liver and modulates the TLR4 signaling pathways (Baumann et al., 2020; Yang et al., 2020). The gut microbiome plays a role in bile acid metabolism by converting primary bile acids into secondary bile acids. In MASLD, this ability is impaired due to reduced abundance of relevant bacteria (Chen and Vitetta, 2020). Microbial modification of bile acids is crucial for maintaining a balanced microbiome, regulating insulin sensitivity and modulating lipid and carbohydrate metabolism, thereby influencing innate immune responses Figure 9 (Jia et al., 2018; Qu et al., 2023). Bile acids also function as signaling molecules through their interaction with nuclear receptors including the farnesoid X receptor (FXR) and G protein-coupled bile acid receptor (TGR5), found in both intestine and liver (de Aguiar Vallim et al., 2013; Jia et al., 2018). These receptors play a central role in regulating bile acid synthesis, bile acid transport and metabolism. Activation of FXR and TGR5 by bile acids influences glucose and lipid metabolism as well as inflammation and immune responses (Leonhardt et al., 2021). Other intestinal microbial metabolites, such as amino acids and choline also involved in the modulation of MASLD (Canfora et al., 2019).

Therapeutic strategies targeting the gut-liver axis in MASLD include various approaches aimed at modulating the gut microbiota, improving gut barrier function and alleviating liver inflammation. One approach is to use probiotics, which are live microorganisms that provide health benefits when consumed in sufficient quantities. Scientific evidence has shown that probiotics can improve liver function and reduce inflammation in patients with MASLD (Li et al., 2016; Xue et al., 2017; Carpi et al., 2022). Probiotics have the ability to regulate the composition of the microbiota, strengthen the function of the intestinal barrier and reduce the translocation of harmful bacteria and their metabolites to the liver (Xue et al., 2017; Konturek et al., 2018). On the other hand, prebiotics considered indigestible food components, selectively stimulate the growth and activity of beneficial microorganisms in the gastrointestinal tract. By promoting the proliferation of beneficial bacteria, prebiotics help rebalance the gut microbiota, contributing to improved liver health (Nagashimada and Honda, 2021; Xing et al., 2022). The synergistic combination of probiotics and prebiotics found in products such as foods, medicines and supplements are called symbiotics (Li et al., 2020). In particular, symbiotics as a combination of probiotics and prebiotics can comprehensively regulate the intestinal microbiota by promoting beneficial bacteria. This results in significant benefits including anti-inflammatory effects, improving intestinal barrier function, maintaining energy homeostasis, regulating lipid metabolism and more (Sarao and Arora, 2017).

Fecal microbiota transplantation (FMT) is a therapeutic approach wherein fecal material from a healthy donor is transferred into the gastrointestinal tract of a recipient individual. FMT reduces inflammation in the colon and initiates the restoration of intestinal homeostasis by activating immune-mediated signaling pathways (Burrello et al., 2018). This results to the production of IL-10 from both adaptive and innate immune cells and ultimately controls intestinal inflammation (Burrello et al., 2018). A recent clinical trial has demonstrated the potential of FMT to improve therapeutic outcomes for MASLD patients. Its effectiveness in the clinical setting appears to be more pronounced in lean MASLD patients than in obese patients (Xue et al., 2022). Conversely, allogeneic FMT did not reduce insulin resistance or liver fat percentage according to magnetic resonance imaging (Craven et al., 2020).

## Conclusion

In this comprehensive bibliometric study, we conducted an analysis of the dynamic research landscape on the complex interplay between gut microbiota and MASH. Our results highlighted several critical aspects of this emerging field. Notably, there was a consistent and robust growth of publications, highlighting the global scientific interest in unraveling the complexity of MASH through a focus on the role of the gut microbiota. Multiorgan crosstalk played a central role in the pathogenesis of MASH. Understanding the complex interplay between the gut and liver is essential for developing effective strategies to treat MASH and its associated complications. Multiorgan crosstalk highlighted the involvement of multiple factors in the development and progression of MASLD/MASH including liver inflammation, alterations in lipid metabolism, mitochondrial dysfunction and gut microbiota dysbiosis. The gut-liver axis played as a central connection between the gut and liver. Dysbiosis of the gut microbiota disrupted intestinal permeability, increased the concentration of portal toxic metabolites, triggered liver inflammation and thus contributed to the development of MASLD and MASH-HCC. Different stages of MASLD have different signatures related to the gut microbiota. The effects of altered gut microbiota in terms of abundance and diversity were mediated by numerous bacterial metabolites including bile acids, butyrate, choline, amino acids and ethanol. The use of probiotics, prebiotics and synbiotics emerged as novel strategies for the treatment of MASLD/MASH. There is evidence that these treatments which focused on the gut microbiota could reverse the gut dysbiosis associated with MASLD/MASH and thereby improve biomarkers of the disease. This strategy diminished liver injury, inflammation and insulin resistance linked to MASLD/MASH. Taken together, these results demonstrated the beneficial effects of probiotics, prebiotics and synbiotics on MASLD/MASH, with their effectiveness depending on the type of treatment, dosage and duration of exposure. Ultimately, additional studies are needed to fully understand the impact of microbiota-based strategies on MASLD/MASH progression. This accumulated knowledge undoubtedly provides the basis for groundbreaking interdisciplinary research efforts aimed at addressing the growing global burden of MASH and its associated metabolic disorders.

## Data Availability

The original contributions presented in the study are included in the article/Supplementary Material, further inquiries can be directed to the corresponding author.
