# Supplementary material for: Global research trends on gut microbiota and metabolic dysfunction-associated steatohepatitis: Insights from bibliometric and scientometric analysis
Source: Front Pharmacol. 2024 Jul 11;15:1390483. doi: 10.3389/fphar.2024.1390483 (PMC11273336; doi:10.3389/fphar.2024.1390483)
Supplement: Supplementary file 1 [file Table1.docx]

**Table S1**| Top 10 authors in gut microbiota and MASH research.

| **Authors** | **Documents** | \| **Citations** \| \| --- \| | **Total link strength** |
| --- | --- | --- | --- | --- |
| Loomba, Rohit | 133 | 16114 | 24692 |
| Sanyal, Arun J | 104 | 15761 | 18453 |
| Byrne, Christopher D | 81 | 6908 | 9910 |
| Younossi, Zobair M | 77 | 12219 | 13159 |
| Wong, Vincent Wai-sun | 75 | 8408 | 15522 |
| Targher, Giovanni | 73 | 8350 | 10384 |
| Harrison, Stephen A | 64 | 8922 | 12840 |
| Nakajima, Atsushi | 61 | 1996 | 5788 |
| Anstee, Quentin M | 57 | 10220 | 12096 |
| George, Jacob | 56 | 9386 | 10510 |

**Table S2 |**Top 10 co-cited authors in gut microbiota and MASH research.

| **Authors** | **Citations** | **Total link strength** |
| --- | --- | --- |
| Younossi, ZM | 5505 | 168270 |
| Chalasani, N | 2377 | 68129 |
| Loomba, R | 2129 | 81654 |
| Angulo, P | 2012 | 62821 |
| Kleiner, De | 1972 | 43214 |
| Targher, G | 1967 | 70362 |
| Eslam, M | 1939 | 58145 |
| Wong, VWS | 1734 | 64472 |
| Younossi, Z | 1610 | 43333 |
| Sanyal, AJ | 1590 | 61120 |
